# KCNQs: Ligand- and Voltage-Gated Potassium Channels

**DOI:** 10.3389/fphys.2020.00583

**Published:** 2020-06-23

**Authors:** Geoffrey W. Abbott

**Affiliations:** Bioelectricity Laboratory, Department of Physiology and Biophysics, School of Medicine, University of California, Irvine, Irvine, CA, United States

**Keywords:** epilepsy, GABA, herbal medicine, hypertension, KCNE, KCNQ2, KCNQ3, KCNQ5

## Abstract

Voltage-gated potassium (Kv) channels in the KCNQ (Kv7) family are essential features of a broad range of excitable and non-excitable cell types and are found in organisms ranging from *Hydra vulgaris* to *Homo sapiens*. Although they are firmly in the superfamily of S4 domain-bearing voltage-sensing ion channels, KCNQ channels are highly sensitive to a range of endogenous and exogenous small molecules that act directly on the pore, the voltage-sensing domain, or the interface between the two. The focus of this review is regulation of KCNQs by direct binding of neurotransmitters and metabolites from both animals and plants and the role of the latter in the effects of plants consumed for food and as traditional folk medicines. The conceptual question arises: Are KCNQs voltage-gated channels that are also sensitive to ligands or ligand-gated channels that are also sensitive to voltage?

## Introduction

Ion channels provide aqueous conduits across cell membranes that allow transmembrane movement of aqueous ions down their electrochemical gradient and across the hydrophobic interior of the lipid bilayer. Although this process is not active transport, it can achieve rates approaching the diffusion limit and also ion selectivity ratios as high as 100:1 even for K^+^ over the smaller but similarly charged Na^+^ (Hille et al., 1999). Ion channels can be categorized using various approaches, such as by their primary sequence (there are many different ion channel gene families organized by sequence similarity), ion selectivity (e.g., K^+^ versus Na^+^) or how they are opened (e.g., by voltage changes or by ligands). Voltage-gated ion channels are best known for their roles in excitable cell processes, including skeletal and cardiac muscle contraction and nervous signaling, functions that demand they respond to changes in membrane potential by opening to allow ion movement that shapes the action potentials in these tissues. Some voltage-gated ion channels also serve vital functions in non-excitable tissues, including in polarized epithelial cells.

Many voltage-gated channels also respond to ligands. It was previously recognized, before the molecular correlates of channels were even identified, that many native voltage-dependent currents are sensitive to small molecules. Indeed, before molecular cloning, pharmacology was the primary method for identifying specific currents. These cases most often involve block of a specific ion channel and consequent inhibition of its current in a process separate from the normal gating processes of the channel or, in some circumstances, augmenting the existing gating processes of the channel, e.g., augmentation of voltage-gated sodium channel inactivation by anti-arrhythmic/analgesic drugs (Viswanathan et al., 2001). However, more recently, we are beginning to understand that some voltage-gated ion channels either require specific endogenous small molecules for their activity or are highly sensitive to a range of endogenous and exogenous small molecules that augment their activity at a given membrane potential. The focus of this review is the KCNQ (Kv7) subfamily of voltage-gated potassium (Kv) channels and the mechanisms of their activation by a range of ligands.

## KCNQ Channel Architecture

**FIGURE 1:**
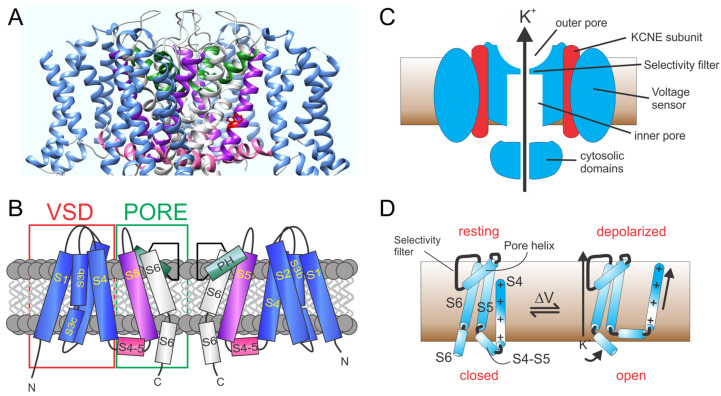
KCNQ channel architecture. **(A)** KCNQ1–KCNQ3 chimeric structural model based on cryo-EM structure of *Xenopus* KCNQ1 (Sun and MacKinnon, 2017). **(B)** Schematic showing membrane topology; domain coloring as above. VSD, voltage sensing domain; PH, pore helix. **(C)** Section through KCNQ1–KCNE complex showing main features. **(D)** Cartoon of voltage gating depicting the S4–S6 portions of a Kv α subunit monomer and the types of conformational shifts that may occur upon membrane depolarization.

Voltage-gated potassium (Kv) channels, including the KCNQs, possess four repeating units, each of which is a separate gene product. The channel forms as a tetramer of KCNQ α subunits. Each unit contains six transmembrane segments with segments 1–4 (S1–S4) forming one voltage sensing domain (VSD) and S5,S6 contributing from each unit to form one quarter of an interlocking pore structure (Figures 1A,B) (Papazian et al., 1987; Tempel et al., 1987; MacKinnon, 1991; Doyle et al., 1998; Long et al., 2005a).

The VSD is the defining feature of voltage-gated ion channels; each VSD contains a transmembrane helix (S4) endowed with periodic basic residues that sense membrane potential (Catterall, 1995). The flexible VSD can, therefore, shift position relative to the membrane electric field in response to membrane potential changes (Figures 1C,D). This conformational shift is transmitted to the pore module via an intracellular linker region, termed the S4–S5 linker in voltage-gated potassium (Kv channels) and other interactions between elements of the pore module and S4 (Bezanilla and Perozo, 2003). Transmembrane segments S1 and S2 within the VSD contain acidic residues that shield or stabilize S4 as it transitions to its activated state upon membrane depolarization (Cuello et al., 2004; DeCaen et al., 2009). Thus, voltage-dependent ion channel gating is electromechanically coupled to the plasma membrane potential (Figure 1D; Long et al., 2005b).

In KCNQ and other K^+^ channels, selectivity over, e.g., Na^+^ ions is achieved by backbone carbonyl oxygen atoms of glycine residues in the canonical GYGD K^+^ selectivity filter motif. This forms a pseudo-hydration shell that accommodates K^+^ perfectly but is too large to properly coordinate Na^+^. Diffusion of K^+^ ions through the K^+^ channel pore requires binding and unbinding of ions to the pore elements (Ranganathan et al., 1996; Doyle et al., 1998; Zhou and MacKinnon, 2003).

Similar to other ion channels, some KCNQ α subunit assemblies also form complexes with one or more non-pore-forming, ancillary subunits (McCrossan and Abbott, 2004; Panaghie and Abbott, 2006; Pongs and Schwarz, 2010). This includes the KCNE subfamily of single-transmembrane domain ancillary subunits (Figure 1C). Each KCNE isoform can regulate more than one type of Kv α subunit (McCrossan and Abbott, 2004), and some Kv α subunit types can each be regulated by more than one KCNE subunit isoform. For example, all five human KCNE isoforms can regulate KCNQ1. The varied functional outcomes of these assemblies permit KCNQ1 to serve a wide variety of functions in both excitable cells, such as ventricular cardiomyocytes, and in non-excitable cells such as gastric and thyroid epithelial cells (Abbott, 2014).

## Regulation of KCNQ Channel Activation by Voltage, Pip_2_ and Ca^2+^

There are five known human *KCNQ* genes in the 40-member Kv α subunit gene family (Abbott, 2014). KCNQ1 is highly studied because of its association with human disease, variety of roles in physiology, and broad functional repertoire. KCNQ2–5 are also highly studied for their roles in the brain (primarily KCNQ2, 3, and 5) (Biervert et al., 1998; Singh et al., 1998; Wang et al., 1998; Tzingounis et al., 2010; Klinger et al., 2011), vasculature (KCNQ4, 5) (Yeung et al., 2007), and auditory epithelium and auditory neurons (KCNQ4) (Kubisch et al., 1999). All the homomeric and heteromeric α subunit-only KCNQ channels studied have been found to be highly sensitive to phosphatidylinositol 4,5-bisphosphate (PIP_2_). Specifically, unlike some other Kv channels that undergo more subtle modulation by PIP_2_, KCNQ channels require interaction with PIP_2_ to pass current (Zaydman et al., 2013). This is because PIP_2_ acts as a cofactor that mediates coupling of the KCNQ VSD with its pore module. Thus, without PIP_2_, VSD activation in response to membrane depolarization does not itself cause pore opening (Zaydman et al., 2013).

This is one example of how we can consider KCNQ channel gating to be ligand- as well as voltage-dependent in much the same way as we consider Ca^2+^-activated K^+^ channels, such as BK, to be both Ca^2+^- and voltage-activated. Interestingly, as for KCNQ channels, BK channel activation is often cast as voltage-initiated but modulated by steady-state intracellular [Ca^2^+]. Yet, in one study designed to mimic physiological conditions, ligand-gating by Ca^2+^ was found to be rate-limiting for BK channel function. It was concluded that BK channels in native systems function primarily as ligand-gated ion channels, activated by intracellular Ca^2+^ (Berkefeld and Fakler, 2013). The Ca^2+^ binding sites on BK channels required for activation are located intracellularly in the Ca^2+^ bowl, located in the C-terminus of the RCK2 domain, and in the RCK1 domain (Lee and Cui, 2010).

For many Kv channels, activation is modeled as a linear process, in which VSD activation must occur before pore opening. In the case of KCNQ channels and, incidentally, also for voltage activation of BK channels, coupling between the VSD and pore module is probably better represented by an allosteric model rather than a linear process. In the allosteric model, the pore is capable of opening without VSD activation, but VSD activation increases the likelihood of pore opening (Zaydman and Cui, 2014). Studies of KCNQ1 have shown that pore opening does not require activation of all four VSDs (Osteen et al., 2012). In addition, we found that, despite the pore of a KCNQ1 mutant (F340W) (in complexes with KCNE1) being locked open, voltage-sensitive inactivation that required S4 movement still occurred, providing additional evidence for non-linear coupling (Panaghie et al., 2008).

It is likely that PIP_2_ is required for allosteric coupling between the activated VSD and the open pore based on studies in KCNQ1 (Zaydman et al., 2013), KCNQ2, and KCNQ3 (Choveau et al., 2018). PIP_2_ probably does not open the pore directly; i.e., effects of coupling alone can explain its obligate role in KCNQ channel opening (Zaydman et al., 2013), but one can still consider KCNQ gating as ligand-dependent as without PIP_2_ the voltage-dependent gating process cannot occur.

PIP_2_ regulation of KCNQs is thought to serve an essential role in control of cellular excitability in tissues expressing KCNQs. For example, PIP_2_ depletion via activation of muscarinic acetylcholine receptors is thought to be a major mechanism for the excitatory action of acetylcholine. By depleting PIP_2_, neuronal M-current (generated primarily by KCNQ2–KCNQ3 heteromers) is inhibited, favoring neuronal depolarization and increased action potential frequency. As KCNQ2–KCNQ3 heteromers are located in key positions, such as the axon initial segment and in nodes of Ranvier, they can act as gatekeepers for action potential propagation (Brown and Adams, 1980; Klinger et al., 2011).

**FIGURE 2:**
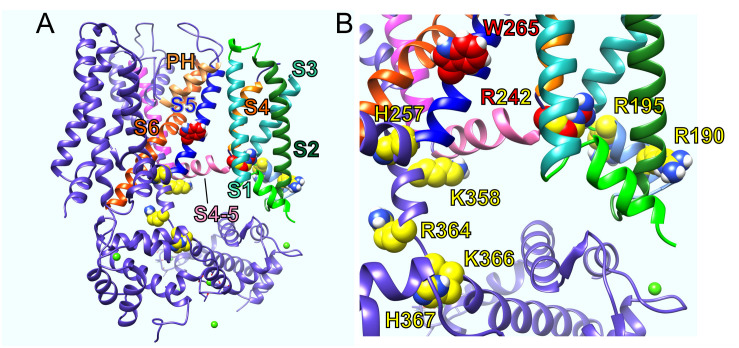
KCNQ residues influential in effects of PIP_2_, GABA, and metabolites. **(A)** KCNQ1–KCNQ3 chimeric structural model showing residues important for effects of PIP_2_ (yellow) and GABA and metabolites (red). PH, pore helix. **(B)** Close-up of residues in A with human KCNQ3 residue numbering.

Various structure–function studies have provided a picture of the likely sites at which PIP_2_ binds and/or acts allosterically on KCNQ channels to exert its strong influence on channel activation. The sites that coordinate functionally relevant interactions of PIP_2_ are clustered in or near the S2–S3 and S4–S5 linkers and at or near the junction between S6 and the C-terminal domain (Kim et al., 2017; Choveau et al., 2018) (Figures 2A,B). This clustering makes perfect sense for the proposed role of PIP_2_ in coordinating bidirectional coupling between the VSD and the pore.

KCNQ channels are also sensitive to Ca^2+^ by virtue of calmodulin (CaM), which is an essential ancillary subunit for KCNQ channels (Gamper et al., 2005; Ghosh et al., 2006; Shamgar et al., 2006). CaM binds to the proximal C-terminus of KCNQ1 and is thought to compete with PIP_2_ to stabilize the KCNQ1 open state; this also occurs in physiological relevant complexes in which KCNQ1-CaM co-assembles with the KCNE1 ancillary subunit to form I_*Ks*_ complexes (see below) (Tobelaim et al., 2017). It is thought that increases in cytosolic Ca^2+^ can compensate for receptor-mediated depletion of PIP_2_ via an interaction between the calcified N lobe of CaM and the KCNQ1 proximal C-terminus (at Helix B) that mimics or substitutes for the KCNQ1-PIP_2_ interaction to curtail *I*_Ks_ current inhibition (Tobelaim et al., 2017). Increased cytosolic Ca^2+^ and consequent CaM calcification may also alter the manner in which CaM binds to KCNQ channels based on experiments with KCNQ2 such that calcified CaM displaces PIP_2_ by altering the binding site and channel affinity for PIP_2_ (Kosenko and Hoshi, 2013). Thus, KCNQ channel gating is also Ca^2+^-sensitive, with Ca^2+^ binding to calmodulin displacing and/or substituting for PIP_2_ to favor opening at more negative membrane potentials. Interestingly, CaM is also required for KCNQ2 exit from the endoplasmic reticulum, and this also, therefore, ensures that even KCNQ2 trafficking is ligand (Ca^2+^)-dependent (Alaimo et al., 2009).

## Regulation of KCNQ Channel Activation by Kcne Subunits

Channels formed by KCNQ1 in complexes with the single transmembrane domain KCNE1 ancillary subunit are essential in the heart and auditory system. The most striking result of KCNQ1–KCNE1 co-assembly is that KCNE1 greatly slows KCNQ1 activation (Yang et al., 1997; Sesti and Goldstein, 1998). Among the mechanistic models proposed for this effect, some suggest that KCNE1 slows S4 movement (Nakajo and Kubo, 2007; Ruscic et al., 2013), and others support direct slowing of KCNQ1 pore opening by KCNE1 (Rocheleau et al., 2006) or that KCNE1 imposes the need for movement of multiple voltage sensors before KCNQ1 channels open unlike what is thought to be the case for homomeric KCNQ1 (Osteen et al., 2010, 2012).

**FIGURE 3:**
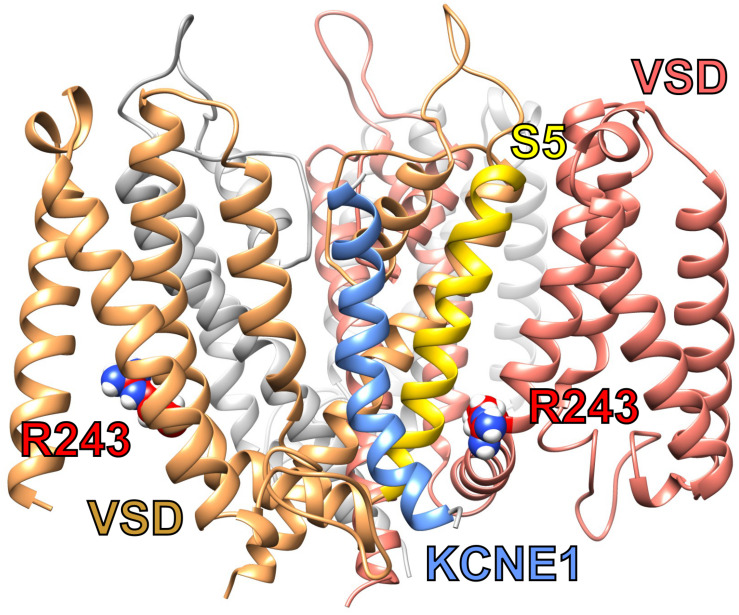
Predicted structure of KCNQ1-KCNE1 complex. KCNQ1–KCNE1 structural model showing predicted position of KCNE1 (blue between two KCNQ1 subunits (sand and salmon) and close to S5 (yellow) and R243 (red) or one subunit. Plotted from model coordinates made available in Kang et al. (2008).

To modulate KCNQ1 in this manner, KCNE1 probably lies in a groove close to both the pore module and VSD (Figure 3). Essential for ventricular repolarization and for endolymph secretion in the inner ear, KCNQ1–KCNE1 channels also require PIP_2_ for activation and are a hundredfold more sensitive to PIP_2_ than are homomeric KCNQ1 channels. The proposed binding site for PIP_2_ abuts regions considered important for KCNE1 regulation of KCNQ1, helping to explain the considerable influence of KCNE1 on KCNQ1 PIP_2_ sensitivity (Li et al., 2011).

KCNQ1 can be converted to a constitutively open channel exhibiting relatively little voltage dependence across the physiological voltage range by forming heteromeric complexes with either KCNE2 (in gastric parietal cells) or KCNE3 (in intestinal epithelium) (Lee et al., 2000; Schroeder et al., 2000; Tinel et al., 2000; Roepke et al., 2006). To perform their function in gastric glands, i.e., provide a K^+^ efflux pathway from parietal cells to supply the gastric H^+^/K^+^-ATPase with luminal K^+^ ions so that it can secrete protons into the stomach lumen, KCNQ1–KCNE2 channels must exhibit two properties. First, they must stay open and non-inactivated at weakly negative voltages (probably largely between −40 and −20 mV in parietal cells); second, they must continue to function efficiently while withstanding the highly acidic extracellular environment of the gastric pits (pH 2–3).

The constitutive activation is probably achieved by KCNE2 favoring the activated conformation of the VSD, a phenomenon that has been studied in much greater detail for KCNQ1–KCNE3 channels because their greater macroscopic currents facilitate electrophysiological and mutagenesis-based structure function studies (Abbott, 2015, 2016). Homomeric KCNQ1 channels are inhibited by extracellular H^+^ ions, manifested as a positive shift in the voltage dependence of activation and slower activation. KCNQ1–KCNE2 current, in contrast, is augmented by low extracellular pH. This property is endowed by the KCNE2 extracellular N-terminus and neighboring region of the transmembrane domain and is not a general property of constitutively open KCNQ1 channels because KCNQ1–KCNE3 channels are largely voltage-independent (see below) yet insensitive to pH (Heitzmann et al., 2004, 2007).

Unlike KCNE2, KCNE3 permits KCNQ1 to pass macroscopic outward currents at levels equivalent to or greater than those generated by homomeric KCNQ1 (Schroeder et al., 2000). KCNE3 favors constitutive activation of KCNQ1 by locking the voltage sensor in its activated conformation rather than by directly locking the pore open independent of the voltage sensor (Nakajo and Kubo, 2007; Panaghie and Abbott, 2007). The McDonald lab discovered that residues in the KCNE3 transmembrane domain are essential for KCNQ1–KCNE3 constitutive activation (Melman et al., 2001, 2002). We subsequently identified additional KCNE3 residues at the membrane-proximal region of the extracellular portion of KCNE3 (D54, D55), which interact with KCNQ1–S4 as part of the constitutive activation process (Choi and Abbott, 2010). It is not yet entirely clear how PIP_2_ regulates gating of constitutively active KCNQ1–KCNE2 or KCNQ1–KCNE3 channels, but co-assembly of KCNQ1 with KCNE3 protects the channel from the inhibition observed for homomeric KCNQ1 upon sphingomyelinase removal of lipid head groups from the plasma membrane (Choi and Abbott, 2010). Furthermore, in recent cryo-electron microscopy–resolved structures in the presence and absence of PIP_2_, KCNE3 appeared to be locking open the voltage sensor as we previously concluded (Panaghie and Abbott, 2007), but in the absence of PIP_2_, the pore still appeared to be closed (Sun and MacKinnon, 2020). Further electrophysiological analyses are necessary to confirm these findings, but it appears that even KCNQ1–KCNE3 channels require PIP_2_ for VSD-pore coupling and pore opening, at least in the conditions used for structural analysis.

## Activation of KCNQ Channels by Direct Binding of Neurotransmitters and Metabolites

Neuronal KCNQ channels (KCNQ2-5 isoforms) each contain a conserved Trp residue in their S5 segment that confers sensitivity to the anticonvulsant, retigabine (Tatulian et al., 2001; Schenzer et al., 2005). Binding of retigabine to KCNQ2–5 isoforms negative-shifts their voltage dependence of activation. At a given voltage, wash-in of retigabine increases KCNQ2–5 current without the need to change the voltage. Thus, although KCNQ channels are highly voltage-sensitive, they can be “activated” at a given voltage by a ligand (retigabine) that requires (and likely binds to) the S5 Trp (W265 in KCNQ3, for example). KCNQ3 is the most highly retigabine-sensitive KCNQ channel, but KCNQ2, 4, and 5 each also respond. KCNQ1 is not activated by retigabine because KCNQ1 lacks the S5 Trp (Schroder et al., 2001).

**FIGURE 4:**
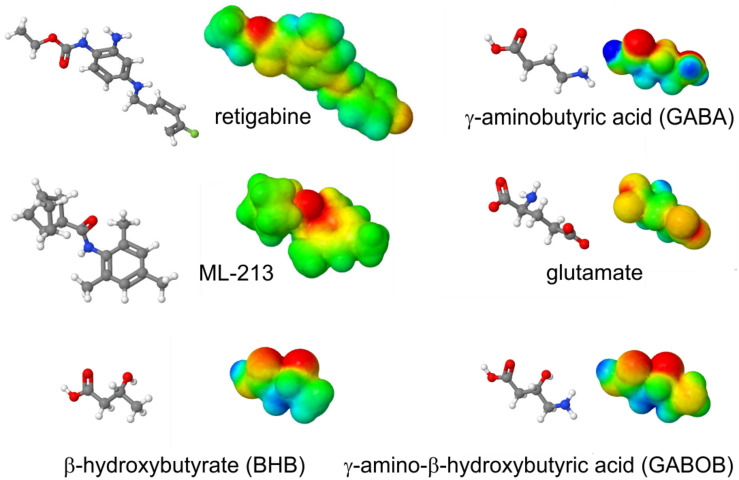
Structures and calculated electrostatic surface potentials of KCNQ activators. Structures and calculated electrostatic surface potentials of KCNQ activators as indicated; red = negative, blue = positive extremes. Plotted using Jmol.

**FIGURE 5:**
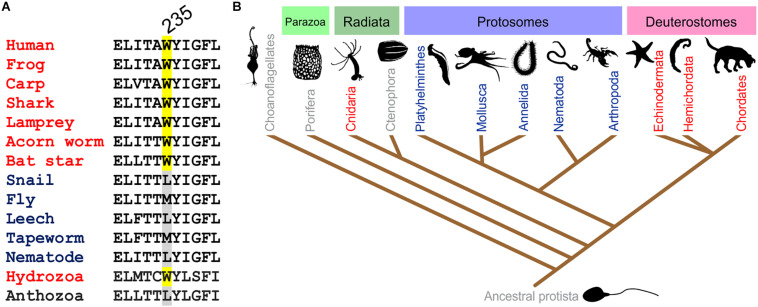
Conservation of the KCNQ5 S5 Trp. **(A)** Sequence alignment of the GABA-binding S5 Trp-containing portion of KCNQ5 or the closest ortholog in various organisms. Adapted with permission from Manville et al., 2018. **(B)** Evolutionary relationships of organisms showing representation of KCNQ channels in major animal clades. Red, organisms contain multiple KCNQs, including one or more with the GABA-binding S5 Trp; blue, one KCNQ, lacking the GABA-binding S5 Trp; gray, no KCNQ. From Manville et al. (2018) used with permission.

**FIGURE 6:**
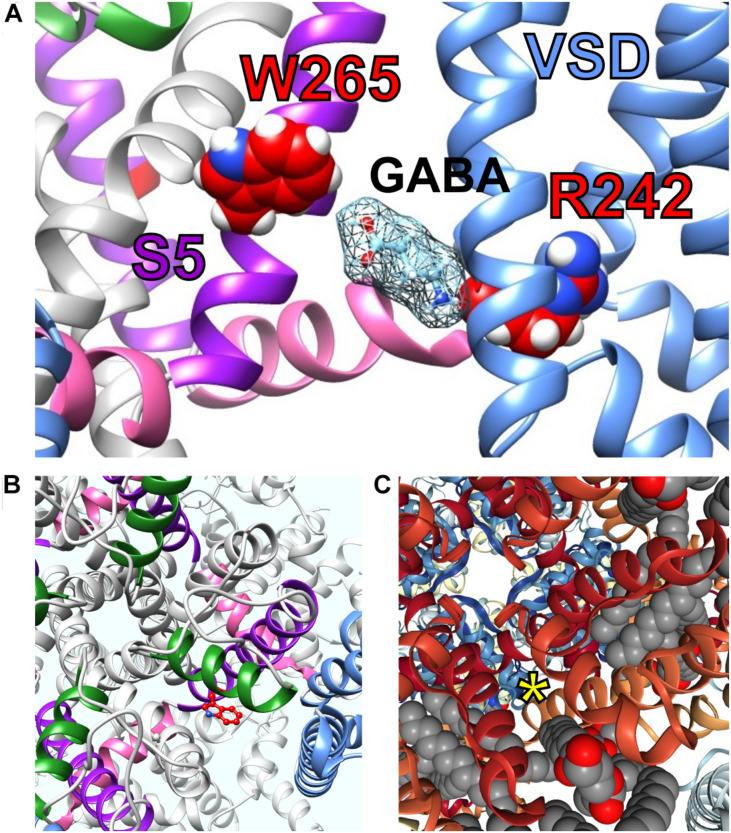
Predicted KCNQ GABA binding site and accessibility. **(A)** SwissDock prediction of GABA binding pose in KCNQ3. VSD, voltage sending domain. **(B)** Extracellular view of KCNQ3-W265 (red) showing possibility access site from outside the cell. Purple, S5; blue, VSD, pink, S4, S5 linker; green pore helix. From Manville et al. (2018) used with permission. **(C)** Similar view as in panel B, but in the Kv1.2–Kv2.1 “paddle-chimera” structure (Long et al., 2007) showing S5 accessibility (*) despite lipid presence (lipids shown in gray/red). Adapted from Manville et al. (2018) with permission.

The S5 Trp is thought to favor binding of (and is certainly required for activation by) molecules such as retigabine because they contain a negative electrostatic surface potential centered on a carbonyl group (Kim et al., 2015) (Figure 4). The S5 Trp has been conserved in evolution for about 600 million years and is present in the neuronal KCNQ-type channels of essentially all deuterostomes and also some of the Cnidaria but absent in protostomes (the genomes of which contain a KCNQ channel that lacks the S5 Trp) (Manville et al., 2018) (Figures 5A,B). Hypothesizing that the S5 Trp is conserved to bind an endogenous ligand, we began looking for ligands with similar chemical properties to retigabine. This led to the discovery that γ-aminobutyric acid (GABA), which also possesses a carbonyl group with associated predicted negative electrostatic surface potential (Figure 4), binds directly and S5-Trp dependently (Figure 6A) to KCNQ2, 3, 4, and 5, but not KCNQ1 (which lacks the S5 Trp) (Manville et al., 2018).

An arginine situated between the voltage-sensing S4 segment and the intracellular linker region that connects S4 to S5 (S4–S5 linker) is also required for GABA binding (Manville et al., 2019) (Figure 6A). This dependence, together with docking studies, suggest a surprisingly deep binding site for GABA, but the site appears to be accessible from the extracellular side (Figures 6B,C). The S4–S5 linker arginine (R242 in KCNQ3) is also in the region that coordinates binding and/or the functional effects of PIP_2_ (Kim et al., 2017). However, activation of KCNQ2/3 by GABA was unaffected by depletion of PIP_2_, either by activation of muscarinic acetylcholine receptors or by wortmannin application (Manville et al., 2018). It is, therefore, possible that GABA works differently from retigabine in that it may not be as dependent on PIP_2_ for coupling of the VSD to the pore for activation or perhaps helps replace PIP_2_ in this role. Interestingly, the S4–S5 linker arginine residue important for PIP_2_ effects on KCNQs, (i.e., KCNQ3–R242, KCNQ5–R212) is also required for GABA effects and, specifically, for binding. We know this because, unlike the large majority of studies examining Kv channel pharmacology, which rely solely on functional effects to infer binding, we directly quantified binding of tritiated GABA to wild-type versus R212A–KCNQ5 (Manville et al., 2019). Given the dual role of this residue in binding of GABA and probably PIP_2_, it is difficult to ascertain the role of PIP_2_ in coordinating GABA effects by mutation of this residue. Future studies could involve more comprehensive depletion of PIP_2_ and then application of GABA followed by quantification of binding and functional effects. The *K*_d_ for GABA binding to KCNQ3 expressed in *Xenopus* oocytes is 126 nM (Manville et al., 2018), which is close to the tonic GABA level in the brain, reported at 160 nM (Santhakumar et al., 2006; Lee et al., 2010). This could mean that neuronal KCNQ channels are equipped to sense changes in tonic GABA levels.

Electrophysiological recordings of cloned channels in *Xenopus* oocytes and CHO cells and endogenous M-current in PC12 cells and mouse dorsal root ganglia (DRG) neurons showed that KCNQ3 and 5, but not KCNQ2 or 4, are “activated” by sub-micromolar to micromolar levels of GABA, making these channels more sensitive than many canonical GABA receptors. GABA negative-shifts the voltage dependence of KCNQ3 and KCNQ5 activation and also that of heteromeric KCNQ2/3 channels, the main molecular correlate of neuronal M-current. At a given voltage (e.g., −60 mV), wash-in of GABA is observed as an increase in activity with immediate onset that peaks at roughly 3 min. Thus, at a constant voltage, KCNQ channels possessing the S5 Trp can be “ligand-activated” by GABA in much the same way as canonical GABA_A_ receptors (albeit without inactivation or desensitization as is observed for GABA_A_ receptors) (Manville et al., 2018).

As GABA is the primary inhibitory neurotransmitter in metazoans, its activation of neuronal KCNQ channels makes physiologic sense, at least in broad terms; i.e., activation of M-current in a neuron tends to reduce excitability of that neuron. The structurally related excitatory neurotransmitter, glutamate, lacks the predicted surface negative surface potential and does not activate KCNQ2/3 channels even at millimolar concentrations (Manville et al., 2018). In addition, mutation of the S5 Trp to a leucine removes the ability of GABA to activate KCNQ2/3 channels and also greatly reduces GABA binding (quantified using tritiated GABA radioligand binding studies in *Xenopus* oocytes expressing KCNQ2/3) (Manville et al., 2018). The inhibitory neurotransmitter, glycine, which does not exhibit a strong surface negative electrostatic potential coupled to a carbonyl group, also did not activate KCNQ2/3 even at millimolar concentrations. However, modification of glycine to add these properties, e.g., by adding specific types of fluorophenyl ring, enabled glycine derivatives to bind to and selectively activate different KCNQ channel isoforms (Manville and Abbott, 2019b).

GABA binds directly to the pore module of KCNQ2-5 α subunits yet only activates channels containing KCNQ3 or KCNQ5. Together with the binding data described above, this suggests that the S5 Trp is required for binding but that other thus-far unidentified residues can influence whether binding leads to activation. What could be the physiological purpose, if any, of GABA binding to specific KCNQ subunits but not opening them? One possibility is that this is so that other gating modifiers can influence whether GABA binding leads to activation in a context-specific manner (see section on interaction with solute transporters below). Another potential explanation is that GABA binding to channels that it does not activate exerts effects because it influences whether other active ligands can enter the GABA binding pocket on KCNQ channels. Yet another possibility is that binding of GABA to, e.g., KCNQ2 when in KCNQ2/3 heteromers does contribute to activation or modulates activation.

Aside from GABA, related endogenous metabolites β-hydroxybutyric acid (BHB) and γ-amino-β-hydroxybutyric acid (GABOB) also activate KCNQ3, KCNQ5, and KCNQ2/3, and they also require the S5 Trp (Manville et al., 2018, 2020). GABOB and BHB each also exhibit predicted negative electrostatic surface potential centered on a carbonyl group (Figure 4). GABOB is present endogenously in mammalian brain (Hayashi, 1959) and has also been utilized as an anticonvulsant drug outside the United States although it is low potency and more effective when used together with another anticonvulsant (Chemello et al., 1980; Swiss Pharmaceutical Society, 2000). GABOB is a partial agonist of GABA_B_ receptors and a GABA_A_ receptor agonist, and we recently found that GABOB is a high-potency partial agonist of KCNQ2/3 channels. GABOB is capable of competing out retigabine, GABA, and BHB from the KCNQ channel GABA binding pocket, reducing their inhibitory effects on excitability both *in vitro* and *in vivo* (Manville et al., 2018, 2020).

BHB is among the first and most important ketone bodies produced when mammals fast or adopt a ketogenic diet. Induction of ketosis has shown promise as an anticonvulsant approach, particularly in children with refractory epilepsy. Although there are several hypotheses for the antiepileptic mechanism of ketosis, BHB levels have been positively correlated with the efficacy of this therapeutic approach (Gilbert et al., 2000). BHB can reach 4 mM in the serum of children on a ketogenic diet and 1 mM in the brain (Pan et al., 2000). BHB activates KCNQ2/3 channels with an EC_50_ of 1 μM; therefore, levels observed in children in ketosis are well within the potential therapeutic range if KCNQ2/3 activation is part of this action. BHB was highly effective as an acute anticonvulsant protective agent in the mouse pentylene tetrazole chemoconvulsant seizure assay, and co-administration of an excess of GABOB reduced the efficacy of BHB in this model (and also reduced BHB activation of KCNQ2/3 *in vitro*) (Manville et al., 2018, 2020). Given this evidence, it is feasible that neuronal KCNQ channels sense the balance between GABA, BHB, GABOB, and other endogenous metabolites to regulate neuronal excitability.

Results from voltage-clamp fluorometry experiments suggest that retigabine binding to the KCNQ3 pore module stabilizes both the open state of the pore module and the activated step of the VSD, resulting in greatly slowed channel closing or deactivation under basal PIP_2_ conditions (Kim et al., 2017). However, when PIP_2_ interaction with the channel was disrupted, either by mutation or by PIP_2_ depletion, the communication between pore module and VSD was greatly disrupted. VSD conformation, as inferred by a greatly increased fluorescence signal from the PIP_2_-disconnected VSD, was highly perturbed although the VSD appeared still able to respond to voltage, and its voltage dependence was still hyperpolarized by retigabine (Kim et al., 2017).

Voltage clamp fluorometry studies of GABA activation of KCNQs have, to our knowledge, thus far not been reported. However, ionic permeability series experiments demonstrated that the relative permeability of Na^+^ and Cs^+^ compared to that of K^+^ is increased by GABA activation of KCNQ3 and KCNQ5 channels but not by binding to KCNQ2 or KCNQ4 channels (which does not result in activation). This suggests that GABA binding to KCNQ3 and KCNQ5 induces a conformational change in the pore module that is not observed upon solely voltage-dependent activation in the absence of GABA (Manville and Abbott, 2020b). GABA activation of KCNQs can be observed as a negative shift in the voltage dependence of activation when plotting an I/V relationship or at a given voltage as an increase in current upon GABA wash-in. Combined with the evidence of a novel pore conformation upon GABA binding only when activation follows, GABA potentiation of KCNQ channel current can be viewed as a ligand-gating process, albeit in a channel that is also sensitive to voltage, analogous to the situation in BK channels with Ca^2+^ and voltage.

## Activation of KCNQ Channels by Direct Binding of Plant Metabolites

Several plant metabolites are now known to bind in a similar location to GABA within KCNQ channels but with some notable differences discussed below. In some cases, KCNQ activation by plant compounds provides a plausible molecular mechanistic basis for the purported benefits of certain botanical folk medicines.

**FIGURE 7:**
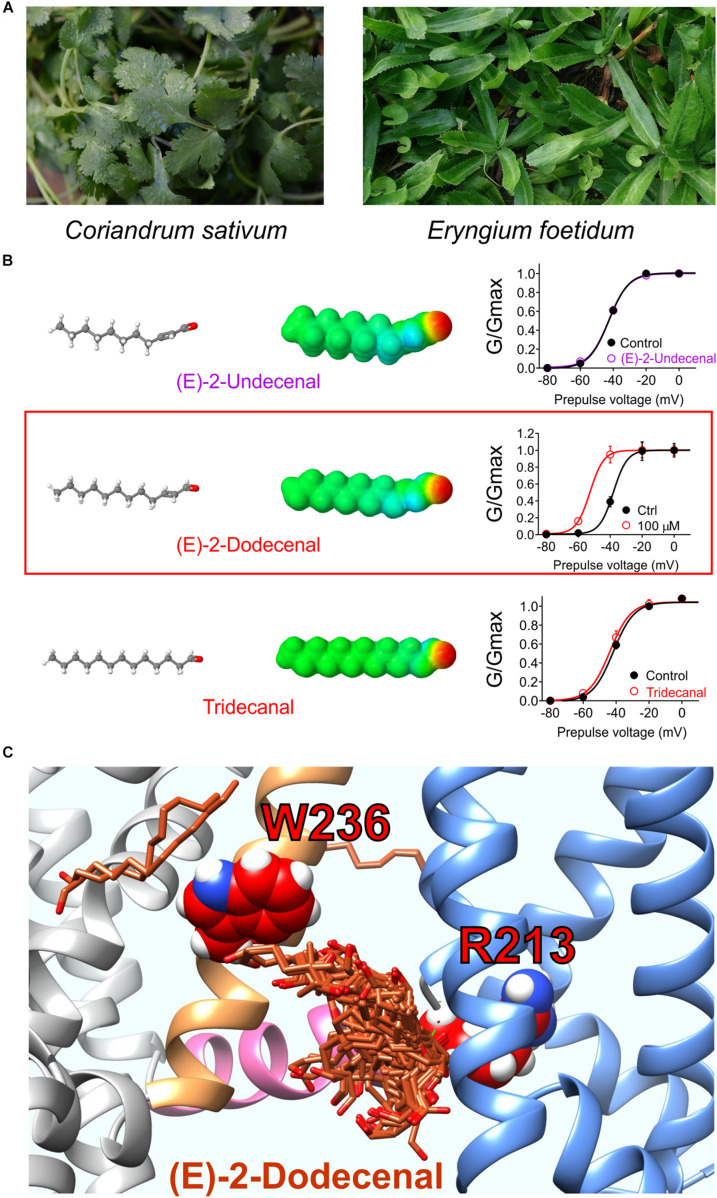
Molecular basis for cilantro anticonvulsant effects. **(A)** Images of cilantro (left) and culantro (right). Required photo permissions: https://creativecommons.org/licenses/by/2.0/; https://commons.wikimedia.org/wiki/File:Culantro_(Eryngium_foetidum)_6.jpg. **(B)** Left, structures and Jmol surface electrostatic potential plots of three structurally similar fatty aldehydes; right, their corresponding effects on KCNQ2/3 G/V relationships (quantified from tail currents). Adapted from Manville and Abbott (2019a) with permission. **(C)** SwissDock prediction of E-2-dodecenal binding poses in KCNQ2. Blue, VSD; sand, S5; pink, S4, S5 linker.

Cilantro (*Coriandrum sativum*) probably originated in Iran (Batmanglij, 2018) and grows wild throughout Western Asia and Southern Europe. Cilantro has been consumed by humans for thousands of years; it was likely cultivated by the Ancient Egyptians and was discovered in the early Neolithic archeological site in Nahal Hemar Cave in Israel (Cumo, 2013). Cilantro and culantro (*Eryngium foetidum*), which has a similar but stronger taste than cilantro, have been used as traditional folk medicines in regions including Central and South America and the Caribbean (Figure 7A). The plants are used to treat a wide variety of disorders, including seizures (culantro is also referred to as fitweed) (Simon and Singh, 1986), and for their purported analgesic, anti-diabetic, and anti-inflammatory properties (Sahib et al., 2013). Each of these species contains several fatty aldehydes that give them the striking citrus-like cilantro/culantro smell and taste.

A 1:100 dilution of aqueous solution obtained by evaporating off the methanol from a methanolic extract of cilantro leaf activated KCNQ2/3 channels expressed in *Xenopus* oocytes by negatively shifting the voltage dependence of activation. Screening of nine compounds previously found by others to be the most highly represented compounds in methanolic cilantro leaf extracts revealed a single compound that activated KCNQ2/3. The compound, E-2-dodecenal, activated KCNQ2 and KCNQ5 preferentially with lesser effects on KCNQ1 and KCNQ4 and no effect on KCNQ3, and KCNQ2/3 channels were highly sensitive (EC_50_ of 60 nM). E-2-dodecenal, also known as eryngial, is a fatty aldehyde; other fatty aldehydes with one more or one less carbon did not activate KCNQ2/3 even at millimolar quantities, indicating surprising selectivity among these compounds (Manville and Abbott, 2019a) (Figure 7B). E-2-dodecenal exhibits a predicted carbonyl-centered negative electrostatic surface potential and was predicted by SwissDock (Grosdidier et al., 2011a, b) docking studies to bind in a pocket near the KCNQ2–W236 residue in S5 but closer to KCNQ2–R213, which, unlike the S5 Trp, is present in the entire KCNQ family (hence, KCNQ1 was also activated by E-2-dodecenal). KCNQ2–R213 lies at the hinge between S4 in the VSD and the S4–S5 linker region. Consistent with docking studies (Figure 7C), in mutagenesis studies, the S5 Trp and the S4–S5 Arg were both required for E-2-dodecenal effects in KCNQ2/3 channels; in KCNQ1, the S4–S5 Arg (R243) was influential but not essential, and it was more important in KCNQ1–KCNE1 channels. This suggests that the requirement for the S5 Trp and the S4–S5 Arg for binding/activation of small molecules are context-dependent and that some molecules may well bind in this same pocket but with different molecular requirements and to different residues. In mouse PTZ-induced seizure studies, E-2-dodecenal was similarly effective at delaying seizures (Manville and Abbott, 2019a) as cilantro extract in a prior study of PTZ-induced seizures in rats (Karami et al., 2015). The findings were consistent with E-2-dodecenal activation of KCNQ2/3 channels contributing significantly to the known anticonvulsant effects of cilantro and culantro.

Interestingly, a loss-of-function variant of the S4–S5 Arg in human KCNQ2 (R213W) associates with both benign familial neonatal convulsions (Sadewa et al., 2008; Miceli et al., 2013) and the much more severe epileptic encephalopathy (Sadewa et al., 2008; Miceli et al., 2013; Milh et al., 2015). Further, the loss-of-function human KCNQ2–R213N variant is linked to epileptic encephalopathy in a two-generation pedigree in which various family members exhibited asymmetric quadriplegia, lifelong myokomia, and/or tonic-clonic seizures (Weckhuysen et al., 2012; Miceli et al., 2013; Orhan et al., 2014), further emphasizing the importance of this residue in KCNQ2 gating and in epilepsy. R213 lies in a BFNE and epileptic encephalopathy mutation hotspot that stretches from the S4-proximal S4–S5 linker to the extracellular end of S4; similar hotspots are present in S6 and the pore region (Zhang et al., 2020). In human KCNQ1, a mutation of the equivalent residue to histidine (R243H) reduces PIP_2_ affinity and is associated with the cardiac arrhythmia, long QT syndrome (Park et al., 2005). Future analyses are required to determine whether compounds such as E-2-dodecenal can rescue epilepsy-associated KCNQ2–R213 or arrhythmia-associated KCNQ1–R243 mutant channels or if the mutation precludes binding and/or functional efficacy as we observed for KCNQ2–R213A.

**FIGURE 8:**
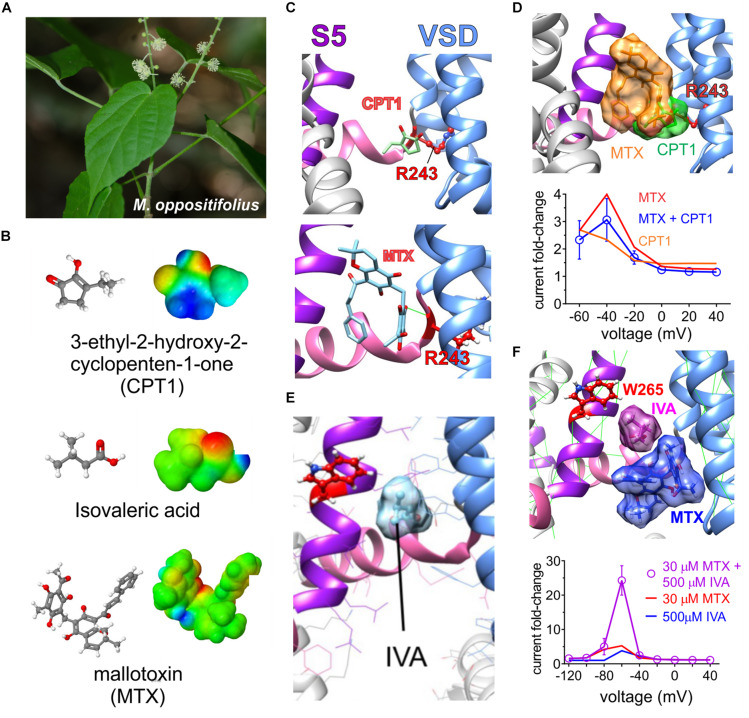
Molecular basis for *Mallotus oppostitifolius* anticonvulsant effects. Images from or adapted with permission from De Silva et al. (2018) and Manville and Abbott (2018). **(A)** Image of *Malloutus oppositifolius*. **(B)** Structures and Jmol surface electrostatic potential plots of three KCNQ-activating compounds from *Malloutus oppositifolius*. **(C)** Predicted binding sites of CPT1 and MTX within KCNQ1. VSD, voltage sensor. **(D)** Upper, predicted overlap of CPT1 and MTX binding sites that would prevent both occupying a single binding pocket in KCNQ1. Lower, Effects of CPT1 and MTX (100 μM) alone or together quantified as KCNQ1 current fold-change versus voltage, showing lack of synergy or summation. **(E)** Predicted binding site of isovaleric acid (IVA) within KCNQ2/3. Red, KCNQ2–W236, or KCNQ3–W265. **(F)** Upper, predicted neighboring binding poses of IVA and MTX binding sites that could allow both to occupy a single binding pocket in KCNQ2 or 3. Lower, Effects of IVA and MTX alone or together quantified as KCNQ2/3 current fold-change versus voltage, showing synergistic effects.

The African shrub *Mallotus oppositifolius* (Figure 8A) is also used as a folk anticonvulsant (Igwe et al., 2016). Previously it was discovered that mallotoxin (a.k.a. rottlerin), a compound found in *Mallotus sp.*, hyperpolarizes the voltage dependence of KCNQ1 channel activation; however, it was at that time concluded to not modulate KCNQ2/3 channels (Matschke et al., 2016). An additional compound in *Mallotus oppositifolius*, 3-ethyl-2-hydroxy-2-cyclopenten-1-one, also activates KCNQ1 via R243 on the KCNQ1 S4–S5 linker (Figures 8B,C), but its effects do not synergize with those of mallotoxin to activate KCNQ1, and they may be unable to both bind in the same pocket (Figure 8D). Activation by *Mallotus* compounds of KCNQ1 alone and in complexes with KCNEs may contribute to its purported therapeutic effects in GI and cardiovascular disorders and diabetes given the known roles of KCNQ1 in these systems (De Silva et al., 2018; Manville and Abbott, 2018).

Unlike the prior study (Matschke et al., 2016), we found that mallotoxin and another compound within *Mallotus oppositifolius*, isovaleric acid (Figure 8B), each activate KCNQ2/3 channels. Mallotoxin and isovaleric acid preferentially activate KCNQ2 over KCNQ3 and appear able to both bind in the same GABA-binding pocket. Mallotoxin apparently binds closer to the S4–S5 Arg, explaining why it can also activate KCNQ1 (De Silva et al., 2018; Manville and Abbott, 2018). Isovaleric acid, which is also found in the commonly consumed herbal supplement valerian root, possesses the characteristic carbonyl-centered negative electrostatic surface potential, is predicted to bind closer to and requires the S5 Trp for activation and, hence, cannot activate KCNQ1 (Figure 8E). Mallotoxin and isovaleric acid are predicted to be able to bind simultaneously in the same binding pocket in KCNQ2/3 channels; accordingly, their activating (and anticonulvsant) effects synergize (Figure 8F) unlike those of MTX and CPT1 (Figure 8D) (De Silva et al., 2018; Manville and Abbott, 2018).

Consistent with docking and mutagenesis studies, GABOB (100 μM) was able to prevent isovaleric acid (500 μM) from activating KCNQ2, suggesting a common binding pocket. Interestingly, as isovaleric acid and mallotoxin preferentially activate KCNQ2, when combined with KCNQ3-preferring retigabine they were so effective that KCNQ2/3 channels were locked open even at −120 mV. *In vitro* studies suggested that combining lower doses of the three could be used as a therapeutic strategy to achieve effective KCNQ2/3 channel opening while minimizing some of the known retigabine-associated toxicity (Garin Shkolnik et al., 2014), but this triple combination has not yet been examined *in vivo* (Manville and Abbott, 2018).

**FIGURE 9:**
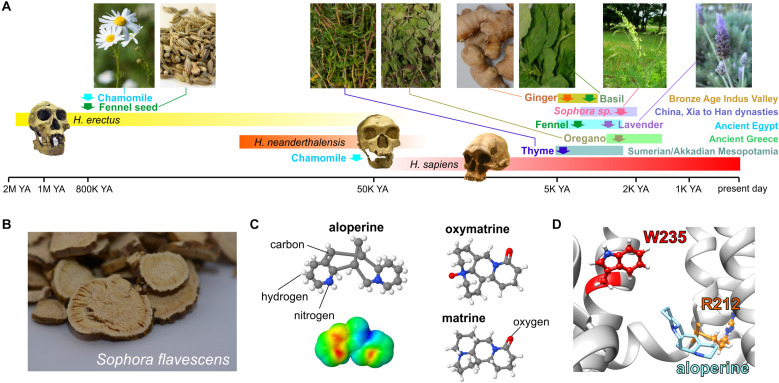
KCNQ5 activation by botanical hypotensive folk medicines. Images from or adapted with permission from Manville et al. (2019). **(A)** Approximate timeline of human use of some of the hypotensive plants discovered to activate KCNQ5. YA, years ago. Required photo permissions: https://creativecommons.org/licenses/by-sa/3.0/; https://en.wikipedia.org/wiki/Chamomile#/media/File:Kamomillasaunio_(Matricaria_recutita).JPG; https://creativecommons.org/publicdomain/zero/1.0/Fennel seed; https://en.wikipedia.org/wiki/Fennel#/media/File:Fennel_seed.jpg; https://creativecommons.org/licenses/by-sa/3.0/Sophora flavescens; https://commons.wikimedia.org/wiki/File:Sophora_flavescens.jpg; https://en.wikipedia.org/wiki/Homo_erectus#/media/File:Homo_Georgicus_IMG_2921.JPG; https://creativecommons.org/licenses/by/2.5/; https://en.wikipedia.org/wiki/Neanderthal#/media/File:Homo_sapiens_neanderthalensis.jpg. **(B)** Dried *Sophora flavescens* root slices. **(C)** Chemical structures of *S. flavescens* compounds showing activity for KCNQ5; *lower left*, surface electrostatic potential plot of aloperine, the most KCNQ5-active compound. **(D)** Predicted binding site of aloperine within KCNQ5.

In the vasculature, channels formed by KCNQ5 (Mackie et al., 2008; Yeung et al., 2008; Mani et al., 2009) alone and/or in complexes with KCNQ4 and the regulatory subunit KCNE4 (Jepps et al., 2011, 2015; Abbott and Jepps, 2016) are influential in setting vascular tone and as such are a potential target for blood pressure–lowering medications. Many botanical folk medicines are thought to help lower blood pressure and are used for this purpose currently. In some cases, this tradition is supported by preclinical and/or clinical evidence (Manville et al., 2019). In a study of 10 such botanical “hypotensive” folk medicines, all 10 activated KCNQ5 channels and strikingly did not activate KCNQ2/3 channels, indicating some degree of isoform specificity. These included very commonly consumed plants, such as ginger, thyme, oregano, basil, marjoram, and fennel as well as herbal remedy staples, such as lavender, chamomile, and *Sophora* species uses in traditional Chinese medicine. Some of these plants have a surprisingly ancient and rich history with respect to their use by human species (Figure 9A; Manville et al., 2019).

One of the plants studied, the traditional Chinese medicine Ku shen (*Sophora flavescens*) (Figure 9B), contains a molecule, aloperine, which isoform-specifically activated KCNQ5 with an EC_50_ of 390 nM. Matrine and oxymatrine, also present in *S. flavescens* (Figure 9C), exerted mild activating effects that moderately augmented those of aloperine but did not synergize with it (Manville et al., 2019). Aloperine requires the S4–S5 linker arginine (KCNQ5–R212) for both binding to (as suggested by competition with tritiated GABA) and activation of KCNQ5 and was predicted to dock close to this residue (Figure 9D; Manville et al., 2019), which is also in or near the predicted PIP_2_ binding site as discussed in the sections on PIP_2_ and GABA above.

**FIGURE 10:**
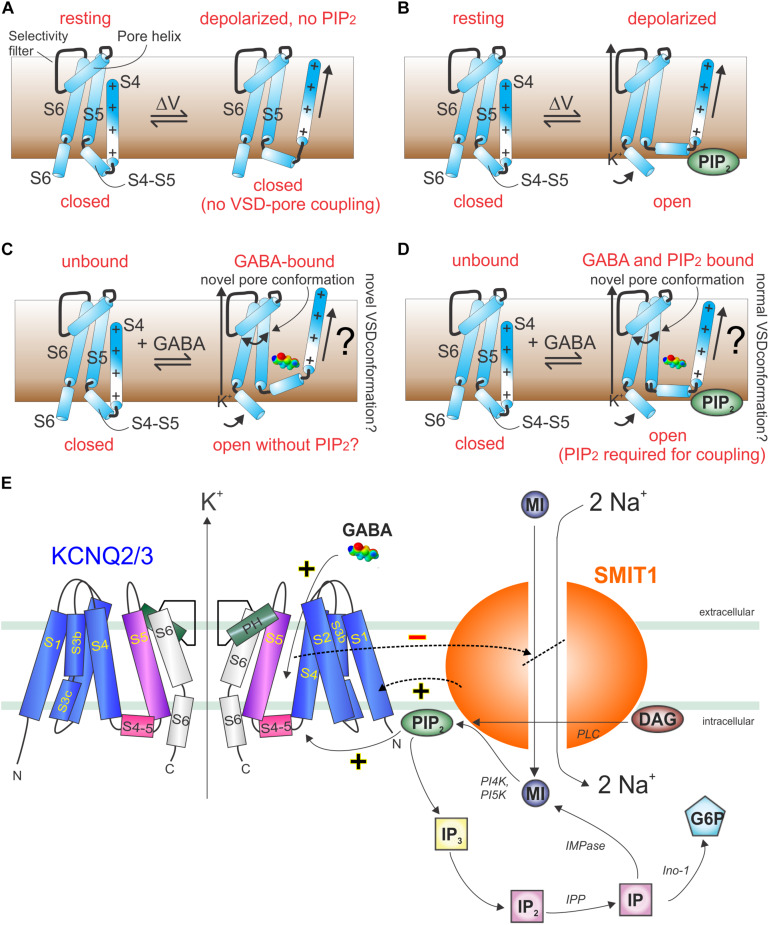
Models of GABA and PIP_2_ signaling in KCNQ and KCNQ2/3–SMIT1 complexes. **(A)** Cartoon showing lack of communication between the KCNQ VSD and pore in the absence of PIP_2_. **(B)** Cartoon showing voltage-dependent activation of KCNQs in the presence of PIP2. **(C)** Cartoon showing hypothetical GABA-induced KCNQ activation in the absence of PIP_2_. **(D)** Cartoon showing hypothetical GABA-induced KCNQ activation in the presence of PIP_2_. **(E)** Cartoon of KCNQ2/3–SMIT1 interactions showing the functional consequences of physical interaction and modulation by small molecules. DAG, diacylglycerol; G6P, glucose-6-phosphate; IMPase, inositol monophosphatase; IP, inositol phosphate; inositol polyphosphatase 1-phosphatase; MI, *myo*-inositol; PI4K, phosphatidylinositol 4-kinase; *PI5K*, phosphatidylinositol-5 kinase; PLC, phospholipase C. From Manville and Abbott (2020b) with permission.

In sum, the above studies strongly suggest that KCNQ channels evolved a binding pocket accessible to neurotransmitters, plant metabolites, and other endogenous and exogenous compounds, many of which can activate KCNQ channels by inducing a novel pore conformation and/or altering coupling between the VSD and pore. Two models are proposed, one requiring PIP_2_ and one not (Figures 10A–D). KCNQ isoform selectivity of the small molecules is varied. Retigabine, for example, activates KCNQ2–5 but not KCNQ1 (because it lacks the S5 Trp) (Schroder et al., 2001); conversely, aloperine is relatively KCNQ5-specific (Manville et al., 2019). Molecules such as aloperine and GABA bind to multiple KCNQ isoforms but only activate a subset; therefore, selectivity is often functional not physical (Manville et al., 2018, 2019). In addition, KCNQs are modulated by a range of different ancillary subunits that can dramatically alter the binding site, selectivity, and/or functional effects of small molecules and must be considered and, where possible, leveraged for increased specificity (Panaghie and Abbott, 2006). Finally, some small molecules, e.g., glycine derivatives that we engineered to activate KCNQs and which rely on the S4–S5 Arg, can exhibit isoform selectivity within the KCNQ family but also activate KCNA1 (but not KCNA2), Kv channels outside the KCNQ family that also bear the S4–S5 Arg (Manville and Abbott, 2020a). Indeed, GABA obviously activates canonical GABA receptors (in addition to KCNQs) (Schofield et al., 1987), and retigabine also activates GABA receptors (Treven et al., 2015). Thus, it is important to recognize that isoform selectivity within a channel family often does not guarantee selectivity in a broader sense—a challenge for drug development in this area.

## Crosstalk Between KCNQ-Solute Transporter Interactions and Ligand Gating of KCNQ Channels

An interesting factor in regulation of KCNQs by small molecules, such as neurotransmitters, metabolites and PIP_2_, is the ability of KCNQs to form reciprocally regulating macromolecular complexes with certain sodium-coupled solute transporters. Members of this class of transporters utilize the downhill sodium gradient from extracellular to intracellular compartments to provide energy powering the uphill transport of solutes, such as sugars, neurotransmitters, and ions, into cells. One such example is the sodium-coupled *myo*-inositol transporters (SMIT1 and SMIT2, encoded by *SLC5A3* and *SLC5A11*), each of which can co-assemble with various KCNQs (Abbott et al., 2014; Manville et al., 2017; Neverisky and Abbott, 2017). *Myo*-inositol is a cyclic polyol that is among the most important osmolytes in mammalian physiology (Berry, 2011). Especially important in the context of this review, it is also a substrate for production of signaling molecules, including phosphatidylinositol phosphates such as PIP_2_ and can transport *myo*-inositol efficiently enough to increase local PIP_2_ concentration and KCNQ-dependently couple osmotic potential to cellular excitability (Dai et al., 2016).

*Myo*-inositol levels in the cerebrospinal fluid (CSF) are tightly regulated. *Myo*-inositol is actively concentrated in the CSF from the blood, primarily across the choroid plexus epithelium, via basolateral uptake through SMIT1 (Spector and Lorenzo, 1975; Guo et al., 1997). SMIT1 is expressed at both the basolateral and apical membranes of the choroid plexus epithelium and can form complexes with KCNQ1–KCNE2 channels at the apical side (Abbott et al., 2014).

KCNQ1–KCNE2 channels inhibit SMIT1 transport activity, reducing *myo*-inositol uptake, when studied in *Xenopus* oocytes (Abbott et al., 2014). SMIT1 is likely sensitive to the conformation of KCNQ1–KCNE2, and the KCNE2 subunit is required for inhibition but requires KCNQ1 to be also be present. KCNQ1-specific inhibitors also inhibit co-assembled SMIT1 activity, suggesting that K^+^ efflux through KCNQ1 helps SMIT1 bring in more substrate, but only when the KCNQ1 conformation is amenable. Apical KCNQ1–KCNE2-SMIT1 channel-transporter complexes may regulate CSF *myo*-inositol composition. Removal of KCNE2 from these complexes may, thus, result in too much *myo*-inositol being removed from the CSF, a hypothesis supported by the finding that *Kcne2*^–/–^ mice have reduced CSF *myo*-inositol, which is associated with increased seizure susceptibility that is corrected by *myo*-inositol mega-dosing (Abbott et al., 2014). We do not yet know why the reduction in CSF *myo*-inositol predisposes to seizures, but it is possible that it causes local imbalances in PIP_2_ and other signaling molecules in a manner that adversely affects ion channel activity, including neuronal KCNQs (Dai et al., 2016; Neverisky and Abbott, 2017).

SMIT1 also physically couples (to the pore module) of KCNQ2 (Manville et al., 2017); SMIT1 and SMIT2 co-assemble with KCNQ2/3 complexes in nodes of Ranvier and axon initial segments in mouse and/or rat brain (Neverisky and Abbott, 2017). SMIT1 alters the pore conformation, gating kinetics, and pharmacology of KCNQ1, KCNQ1–KCNE1, KCNQ2, KCNQ3, and KCNQ2/3 channels (Manville et al., 2017). These effects, including hyperpolarization of KCNQ2/3 channel activation voltage dependence, can occur without the necessity for SMIT1 to be transporting *myo*-inositol; i.e., they occur because of direct physical interaction between SMIT1 and the channels listed (Manville et al., 2017). In addition, however, transport of *myo*-inositol through SMIT1 can increase the substrate for local PIP_2_ production, which can also regulate the KCNQ2/3 channel indirectly (Dai et al., 2016; Neverisky and Abbott, 2017).

GABA and other small molecules that activate KCNQ2/3 channels inhibit co-assembled SMIT1 *myo*-inositol transport activity, both in mouse dorsal root ganglia and when co-expressed in *Xenopus* oocytes (Manville and Abbott, 2020b). This suggests a feedback loop in which GABA activation of KCNQ2/3 reduces the local substrate for PIP_2_ and, thus, eventually inhibits KCNQ2/3, releasing inhibition of co-assembled SMIT1 (Figure 10E; Manville and Abbott, 2020b). Intriguingly, co-assembly of SMIT1 alters KCNQ isoform sensitivity to BHB, such that KCNQ2 is now activated and KCNQ3 not, whereas the reverse is true in the absence of SMIT1 (Manville and Abbott, 2020b). However, GABA isoform sensitivity remains the same. Thus, KCNQ channels act as chemosensors permitting co-assembled transporters to respond to small molecules to which they were previously insensitive (GABA, BHB, retigabine), and conversely, the co-assembled transporter tunes how KCNQs respond to these small molecules (Manville and Abbott, 2020b). When one considers that KCNQ2/3 channels also co-assemble with dopamine and glutamate transporters, the stage is potentially set for previously unexpected regulatory mechanisms, e.g., GABA controlling neuronal uptake of other neurotransmitters via direct binding to voltage-gated channel-transporter complexes (Manville and Abbott, 2019c).

## Conclusion

The physiological importance of specific roles for the signaling mechanisms outlined in this review will become clearer when data are available for mice in which regulation of KCNQs by GABA and related metabolites are disrupted—studies that are ongoing in the author’s laboratory. As discussed for BK channels, KCNQ channels should be considered as channels gated by both voltage and by ligands, and the preponderance of either mechanism is likely context-dependent, specific to a given cell type under specific voltages, ligand concentration ranges, and interacting regulatory proteins that influence all these factors.

As KCNQ channel open probability can be dramatically increased at a given voltage by direct binding of GABA, via induction of a pore conformation that is not observed in activated channels in the absence of GABA (using relative ion permeability as a readout of pore conformation) (Manville and Abbott, 2020b), in this context, the channel can be viewed as ligand-activated. Conversely, in the absence of GABA, KCNQs can open solely in response to membrane depolarization with the sizeable caveat that for this to occur, binding of a different ligand (PIP_2_) near the S4–S5 linker is required to couple VSD movement to the pore module in a manner that actually results in pore opening (Kim et al., 2017).

## Author Contributions

GA wrote the manuscript and prepared the figures.

## Conflict of Interest

The authors declare that the research was conducted in the absence of any commercial or financial relationships that could be construed as a potential conflict of interest.
